# Transverse Myelitis and Guillain-Barré Syndrome Associated with Cat-Scratch Disease, Texas, USA, 2011

**DOI:** 10.3201/eid2409.180008

**Published:** 2018-09

**Authors:** Ramia Zakhour, Pedro Mancias, Gloria Heresi, Norma Pérez

**Affiliations:** American University of Beirut, Beirut, Lebanon (R. Zakhour);; University of Texas UT Health, Houston, Texas, USA (R. Zakhour, P. Mancias, G. Heresi, N. Pérez)

**Keywords:** *Bartonella henselae*, Guillain-Barré syndrome, transverse myelitis, cat-scratch disease, United States, serology, brain lesions, peripheral nerve involvement, bacteria, proteobacteria, zoonoses, Texas

## Abstract

We describe a case of coexisting transverse myelitis and Guillain-Barré syndrome related to infection with *Bartonella henselae* proteobacterium and review similar serology-proven cases. *B. henselae* infection might be emerging as a cause of myelitis and Guillain-Barré syndrome and should be considered as an etiologic factor in patients with such clinical presentations.

A child with lower extremity weakness raises an increasingly complex diagnostic challenge; frequently no etiology is identified (*1*,*2*). We present a case of lower extremity weakness linked to cat-scratch disease (CSD, causative agent *Bartonella henselae* proteobacterium).

In 2011, a 10-year-old girl was transferred to our hospital (UT Health, Houston, Texas, USA) from another hospital, where she had been treated for 2 days for abdominal pain, vomiting, and urinary retention. Seven days before admission to UT Health, she had a left cervical lymphadenopathy. During hospitalization, the patient had urinary retention; lower extremity weakness; worsening headache; neck pain; lower back pain; and a bilateral burning sensation in the wrists, knees, ankles, and feet.

Before her illness, she was healthy and fully immunized; her exposure history only included a cat at home that frequently bit and scratched her. Physical examination revealed a palpable lymph node (3 × 4 cm) at the left cervical lymph node, lower extremity strength of 4 on a 5-point scale (https://www.ncbi.nlm.nih.gov/books/NBK436008/), and decreased deep tendon reflexes. She reported hyperalgesia in her legs.

Peripheral blood cell counts and chemistry test values were within reference ranges. Alanine aminotransferase and aspartate aminotransferase were both mildly elevated (48 U/L [0.8 µkat/L]). A magnetic resonance image (MRI) of the brain showed a focus of increased T2 signal, and an MRI of the spine showed a long centrally located segment of increased T2 signal (Figure). Cerebrospinal fluid (CSF) studies showed a leukocyte concentration of 58 cells/mm^3^ (reference range <10 cells/mm^3^), glucose of 46 mg/dL (nonfasting reference range 45–100 mg/dL), and protein of 55 mg/dL (reference range 15–45 mg/dL). We gave the patient a diagnosis of myelitis and treated her empirically with ceftriaxone and vancomycin, pending CSF culture results. On day 11 of illness, we started administering rifampin and doxycycline for a possible CSD diagnosis; the patient was positive for *B. henselae* IgG (1:152) and IgM (1:160). Increases in *B. henselae* IgG and decreases in *B. henselae* IgM were seen with subsequent serologic tests: day 27 (IgG 1:256, IgM 1:40) and day 41 (IgG 1:512, IgM 1:20). Evaluation for other etiologies included bacteria culture with urine, blood, and CSF samples; CSF latex agglutination for bacterial antigen; virus culture with nasal washes; rapid plasma reagin test; CSF venereal disease research laboratory testing; enterovirus, herpes simplex virus, and mycobacteria PCR of CSF sample; Epstein-Barr virus and cytomegalovirus PCR of serum samples; and *Mycoplasma pneumoniae*, West Nile virus, *Borrelia burgdorferi*, and human T-cell lymphotropic virus I and II antibody testing, all of which were negative for evidence of the respective microbial agents. CSF angiotensin-converting enzyme and IgG levels were in reference ranges. CSF myelin basic protein level (6.4 ng/mL [reference range <1.1 ng/mL]) was elevated. Vitamin B12 and folate levels were in reference ranges, and antinuclear antibody, rheumatoid factor antibody, and dsDNA antibodies were absent.

**Figure:**
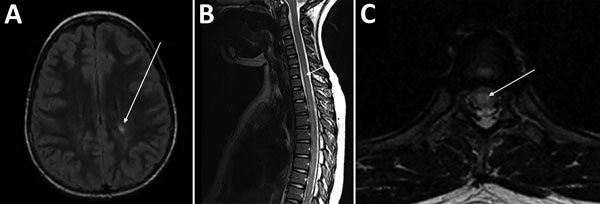
Magnetic resonance images (MRIs) on day 10 of illness in a 10-year-old girl with transverse myelitis and Guillain-Barré syndrome associated with cat-scratch disease, Houston, Texas, USA, 2011. A) Brain MRI. Arrow indicates focus of increased T2 signal in the left posterior periventricular and deep white matter. B) Sagittal spine MRI. Arrow indicates long segment of increased T2 signal centrally located within the spinal cord. C) Axial thoracic spine MRI. Arrow indicates increased central signal within the spinal cord.

The patient completed a 14-day course of doxycycline; rifampin was discontinued after 5 days because of rising liver enzyme levels. By day 34 of illness, the patient’s muscle strength substantially improved, but she continued to have difficulty voiding and severe lower extremity pain. A repeat MRI showed resolution of the increased thoracic spinal cord signal and a new enhancement of the cauda equina nerve roots. Repeat lumbar puncture indicated a leukocyte concentration of 12 cells/mm^3^ (85% lymphocytes [reference range 62% ± 34%]). Nerve conduction studies revealed patchy mixed demyelinating axonal motor and sensory neuropathy. After intravenous immunoglobulin administration for possible Guillain-Barré syndrome (GBS), she showed tremendous improvement, with resolution of urinary retention and a substantial decrease in pain and weakness; 4 months later, she had only residual sensory deficits.

*B. henselae* proteobacterium is transmitted to humans typically through cat scratches or bites (*3*). Neurologic complications, usually self-limited, develop in 0.2%–3.0% of CSD patients (*4*). The first case of CSD with neurologic manifestations was described in 1952. By 1971, ≈40 cases had been reported (*5*), 90% involving encephalitis and a few myelopathy (*6*). The cases of myelopathy had slower recovery courses than those of encephalitis, as well as more residual deficits.

Four other serology-documented CSD-associated myelitis cases (*3*,*4*,*7*) and 1 other GBS-associated *B. henselae* infection (in a 10-year-old girl) (*8*) have been described. Carman et al. reported a case similar to the one we describe: myelitis and GBS in a 12-year-old boy (*9*).

Studies of the efficacy of treatments for CSD-associated neurologic manifestations are lacking, and thus, the optimal regimen and duration of therapy are unknown. However, we suggest that clinicians consider CSD early in disease courses involving neurologic complications; the possibility of GBS, myelitis, or both in the setting of possible CSD should prompt clinicians to initiate antimicrobial treatment early and consider steroid or intravenous immunoglobulin therapy to prevent progression of disease.

This patient had an unusual presentation of CSD, with evidence of myelitis, brain lesions, and peripheral nerve involvement. Although few cases of CSD-associated transverse myelitis and GBS have been described, clinicians should be aware of the existence of this clinical scenario and include it as a differential diagnosis for these 2 syndromes in the pediatric age group.

## References

[1] Marx A, Glass JD, Sutter RW. Differential diagnosis of acute flaccid paralysis and its role in poliomyelitis surveillance. Epidemiol Rev. 2000;22:298–316. 10.1093/oxfordjournals.epirev.a018041 11218380

[2] Centers for Disease Control and Prevention. AFM investigation. 2015 Apr [cited 2018 Jan 2]. https://www.cdc.gov/acute-flaccid-myelitis/afm-surveillance.html

[3] Baylor P, Garoufi A, Karpathios T, Lutz J, Mogelof J, Moseley D. Transverse myelitis in 2 patients with *Bartonella henselae* infection (cat scratch disease). Clin Infect Dis. 2007;45:e42–5. 10.1086/519998 17638185

[4] Salgado CD, Weisse ME. Transverse myelitis associated with probable cat-scratch disease in a previously healthy pediatric patient. Clin Infect Dis. 2000;31:609–11. 10.1086/313986 10987731

[5] Lyon LW. Neurologic manifestations of cat-scratch disease. Report of a case and review of the literature. Arch Neurol. 1971;25:23–7. 10.1001/archneur.1971.00490010033004 5146407

[6] Lewis DW, Tucker SH. Central nervous system involvement in cat scratch disease. Pediatrics. 1986;77:714–21. 3703639

[7] Sendi P, Hirzel C, Bloch A, Fischer U, Jeannet N, Berlinger L, et al. *Bartonella*-associated transverse myelitis. Emerg Infect Dis. 2017;23:712–3. 10.3201/eid2304.161733 28322716 PMC5367412

[8] Massei F, Gori L, Taddeucci G, Macchia P, Maggiore G. *Bartonella henselae* infection associated with Guillain-Barre syndrome. Pediatr Infect Dis J. 2006;25:90–1. 10.1097/01.inf.0000195642.28901.98 16395116

[9] Carman KB, Yimenicioglu S, Ekici A, Yakut A, Dinleyici EC. Co-existence of acute transverse myelitis and Guillain-Barré syndrome associated with *Bartonella henselae* infection. Paediatr Int Child Health. 2013;33:190–2. 10.1179/2046905512Y.0000000044 23930734

